# The construction and evaluation of a prognostic risk score model for HCC based on MPT-related lncRNAs

**DOI:** 10.3389/fonc.2025.1590094

**Published:** 2025-07-28

**Authors:** Zerun Lin, Jianda Yu, Zhijian Chen, Jingyi Chen, Xiaobin Chi, Honghuan Lin, Yongbiao Chen

**Affiliations:** ^1^ Fuzong Clinical Medical College of Fujian Medical University, 900th Hospital of PLA Joint Logistic Support Force, Fuzhou, China; ^2^ The Second Afliated Hospital of Fujian University of Traditional Chinese Medicine, Fuzhou, China; ^3^ Department of Hepatobiliary Surgery, 900th Hospital of PLA Joint Logistic Support Force, Fuzhou, China; ^4^ Department of Clinical Medicine, Fujian Medical University, Fuzhou, China

**Keywords:** HCC, TCGA, lncRNA, MPT, prognostic model

## Abstract

**Background:**

Hepatocellular carcinoma (HCC) is the second leading cause of cancer-related deaths in China. It has a high rate of postoperative recurrence and lacks prognostic markers. In this study, we first analyzed mitochondrial permeability transition (MPT) necrosis-associated long non-coding RNAs (lncRNAs), integrated multi-omics, and constructed a prognostic model. We also revealed the mechanism by which it regulates the immune microenvironment. This provides a new target for targeted therapy in HCC.

**Objective:**

Screening and construction of a prognostic risk score model for MPT-driven necrosis-associated lncRNAs in HCC and exploration of their potential role in HCC.

**Methods:**

Pearson’s correlation analysis, in conjunction with The Cancer Genome Atlas (TCGA) and gene set enrichment analysis (GSEA) databases, was utilized for the identification of lncRNAs associated with mitochondrial permeability transition-driven necrosis. The development of a risk prognostic score for mitochondrial permeability transition-driven necrosis-associated lncRNAs was accomplished through the implementation of one-way regression analysis and Least Absolute Shrinkage and Selection Operator (LASSO) regression analysis. Bioinformatics analysis was performed to validate the prognostic ability and clinical application efficacy of the risk score model and prognostic genes and to explore their biological significance.

**Results:**

MPT-driven necrosis-related lncRNAs (MPTDNRlncRNAs) strongly correlated with HCC were obtained through Pearson’s correlation analysis. Additionally, MPT-driven necrosis-related prognostic lncRNAs were obtained through univariate Cox regression analysis. A new prognostic risk model consisting of three MPTDNRlncRNAs was constructed using LASSO-Cox regression. The model was tested using multiple bioinformatics methods, which suggested that it could significantly differentiate between high- and low-risk groups (p < 0.05) and demonstrated good survival prediction efficacy [area under the curve (AUC) = 0.725]. Differential genes in the high- and low-risk groups were enriched in pathways related to the cell cycle and cellular composition. Combined with immune cell infiltration and immune function scores, these results showed that the patients in the low-risk group had a more significant clinical response to immunotherapy (p < 0.05). Furthermore, the expression level of prognostic genes was verified using the RT-qPCR method on cancerous and paracancerous tissues from HCC patients who underwent HCC resection at our hospital.

**Conclusion:**

The risk scoring model and prognostic genes in this study have been shown to possess satisfactory predictive values, which may prove beneficial for the assessment of risk and the selection of individualized chemotherapy regimens for patients with HCC. A preliminary discussion is presented on the potential biological significance of risk scores in HCC.

## 1 Introduction

Primary hepatocellular carcinoma (HCC) represents a substantial global health concern. Extant literature indicates that in China, HCC has the fourth-highest incidence rate of new cancer cases, the fifth-highest incidence rate, and the second-highest mortality rate. This underscores the significance of HCC as one of the most prevalent malignant neoplasms of the digestive system, representing a substantial threat to the health and wellbeing of the Chinese population (1, 2). The clinical presentation of HCC can vary, manifesting in three distinct pathological forms: HCC, intrahepatic cholangiocarcinoma (ICC), and combined hepatocellular–cholangiocarcinoma (HCC-CCA). The last two forms are regarded as distinct neoplasms. HCC constitutes the majority of these cases (3, 4). Although hepatectomy is widely acknowledged to improve the prognoses of patients with HCC, the risk of postoperative recurrence and metastasis persists. Current clinical prognostic markers, such as alpha-fetoprotein (AFP) and protein induced by vitamin K absence-II (PIVKA-II), are characterized by clear limitations with regard to sensitivity and specificity. Consequently, the development of novel molecular prognostic models is imperative to enhance the precision of HCC diagnosis and treatment. Recent studies have demonstrated that HCC cells gain a survival advantage by remodeling mitochondrial homeostasis. This metabolic adaptive change may contribute to treatment resistance and recurrent metastasis (5).

In the course of investigating the mechanisms underlying HCC recurrence, mitochondrial permeability transition (MPT)-mediated programmed necrosis has emerged as a focal point in research endeavors. Preliminary studies have demonstrated that the HBV X protein (HBx) facilitates the resistance of hepatocellular carcinoma cells to sorafenib by impeding the opening of MPT-related channels (6). A clinicopathologic analysis revealed that abnormally high expression of CypD in HCC tissues was significantly associated with early postoperative recurrence (7). This form of necrosis, which is regulated by MPT, plays a dual role. In the physiological state, it eliminates genetically damaged cells. In the pathological state, it has been demonstrated to promote tumor immune escape by releasing damage-associated molecular patterns (DAMPs) (8, 9). This finding suggests that the disruption of the dynamic equilibrium of MPT may serve as a crucial molecular link between chronic liver injury and the malignant progression of HCC (10).

Long-chain non-coding RNAs (lncRNAs) have been demonstrated to play a pivotal role in epigenetic regulation. These molecules have been observed to influence energy metabolism, cell cycle, and other pathways, thereby contributing to the progression of HCC (11–14). For instance, lncRNA H19 has been demonstrated to promote self-renewal of hepatocellular carcinoma stem cells by regulating Wnt/β-catenin signaling (15), while SBF2-AS1 has been shown to enhance tumor invasiveness through a competing endogenous RNA(ceRNA) mechanism (16, 17). Recent studies have demonstrated that MPT-driven necrosis-related lncRNA (MPTDNRlncRNA) possesses the capacity to modulate mitochondrial membrane potential and influence the sensitivity of hepatocellular carcinoma cells to MPT inducers by selectively targeting miR-365 (18). This finding provides direct evidence for the analysis of the mechanism of HCC recurrence from the MPT–lncRNA regulatory axis.

## 2 Materials and methods

### 2.1 Data collection

The Cancer Genome Atlas (TCGA) database (19), also known as the Cancer Genome Atlas Project, is a comprehensive database that assembles multifaceted cancer genetic information to facilitate research on cancer diagnosis, treatment, and prevention by integrating multidimensional data from multiple cancer types, multiple histologic data, and multiple sample information. The TPM format RNA sequencing data and clinical information of 363 patients with hepatocellular carcinoma (excluding six samples with missing follow-up time) from TCGA-LIHC were obtained from TCGA database. The data were standardized by log2 (value + 1). The MPT-driven necrosis-related gene set (M17902, M3873, and M16257.gmt) was screened based on the gene set enrichment analysis (GSEA) database, and 39 key genes were extracted for subsequent analysis.

A total of 30 pathological tissue samples were retrospectively collected from patients who underwent hepatocellular carcinoma surgery in the Department of Hepatobiliary Surgery, the 900TH Hospital of Joint Logistics Support Force, from September 1, 2022, to September 1, 2024. Each sample consisted of tumor tissue and its paired paraneoplastic tissue. The following inclusion criteria must be met for a patient to be considered eligible for the study: 1) confirmed diagnosis of hepatocellular carcinoma, as determined by postoperative pathologic examination; 2) liver function based on the Child–Pugh grading of grade A or B, indicating the patient’s ability to tolerate the surgical procedure; 3) a radical resection of hepatocellular carcinoma, performed for the first time; and 4) complete follow-up data. The following exclusion criteria were employed: 1) preoperative treatment with targeted and immune therapies, 2) accompanied by severe underlying diseases, 3) perioperative and non-tumor-related deaths, and 4) combined with tumors of other origins. Informed consent was obtained from all patients before surgery.

### 2.2 Screening of MPT-related lncRNAs

We annotated the transcriptomic profiles of LncRNA genes using R packages 'tidyverse' and 'BiocManager', and subsequently constructed a hepatocellular carcinoma-specific MPT necrosis-related gene expression profile along with a standardized LncRNA expression matrix based on the limma package. A screening of co-expressed lncRNAs with a significant association with the MPT necrosis pathway was conducted using Pearson’s correlation (20) coefficients (|cor| > 0.4, p < 0.001) (21). The results were then visualized through the utilization of Sankey diagrams and co-expression networks.

### 2.3 Kaplan–Meier survival analysis

The “survival” function of the R language “survival” package was used to generate the survival curve model, and the ggplot2 package was used to plot the Kaplan–Meier survival curve. The survival differences between the various groups were compared by means of the log-rank test.

### 2.4 ROC analysis

Receiver operating characteristic (ROC) curves were constructed to assess the model’s performance, and the area under the curve (AUC) was calculated to quantify the prediction accuracy. An AUC > 0.6 indicated that the model exhibited a satisfactory ability to predict the patient’s prognosis.

### 2.5 Construction of lncRNA risk score related to MPT-driven necrosis

The samples were randomly divided into training and validation groups, and lncRNAs significantly associated with prognosis were screened using Cox proportional risk regression analysis (p < 0.05). The final lncRNAs were determined by LASSO regression. Risk scores of the HCC patients were calculated using the following formula: $risk\;score=\sum_{i=1}^{n}exp_{i}*\beta_{i}$ . Patients were divided into high- and low-risk subgroups by median to further analyze the relationship between risk score and overall survival. The external validation was executed by employing RNA-seq data and clinical information from the International Cancer Genome Consortium - Liver Cancer - French Cohort (ICGC-LIRI-FR) cohort. Risk scores were calculated identically to TCGA cohort, and survival analysis followed the same statistical protocols.

### 2.6 Real-time quantitative polymerase chain reaction

The primers for LINC00685, GIHCG, MIR210HG, and GAPDH are listed in **Table 1**. The RNA was extracted using the TRIzol method, and the qPCR was detected using the SYBR Green method. The reaction conditions were as follows: initial denaturation at 95°C for 3 minutes, followed by 40 cycles of denaturation at 95°C for 15 seconds and extension at 60°C for 30 seconds. The resulting data were analyzed using the 2^−ΔΔCt^ method.

**Table 1:** Primer sequences of MPT-associated lncRNAs.

LINC00685	F primer (5′–3′)	TGCAAGCTCCAGTCTACCTTC
	R primer (5′–3′)	AAACGGTGGCTACATTTCCG
GIHCG	F primer (5′–3′)	CTTCACAAGCGGTTATCCAGTC
	R primer (5′–3′)	TGGGCCACACTTCATTTCAC
MIR210HG	F primer (5′–3′)	CCACCTCTGGGGACTTCCTA
	R primer (5′–3′)	CTGAAGCGGCAGAAACACAC
GAPDH	F primer (5′–3′)	TTCCTACCCCCAATGTGTCC
	R primer (5′–3′)	GGTCCTCAGTGTAGCCCAAG

MPT, mitochondrial permeability transition; lncRNAs, long non-coding RNAs.

### 2.7 Column line graph construction and multidimensional validation analysis

The construction of a prognostic prediction column chart was based on the Cox proportional risk model. Calibration curves were used to assess the consistency of predicted probabilities with actual observed probabilities. The model prediction efficacy was quantified through the C-index and time-dependent ROC curves (22). Principal component analysis (PCA) was used to downsize the high-dimensional data. The R language scatterplot3d (23) package was used to visualize the results of PCA. The limma package (24) was utilized to identify differential genes (|log2FC| ≥ 1, FDR < 0.05), and the R package “ggplot2” was employed to generate volcano plots of the differentially expressed genes (DEGs) in the database to visualize the disparities in gene expression. Gene Ontology (GO) and Kyoto Encyclopedia of Genes and Genomes (KEGG) enrichment analyses were performed using the “clusterProfiler” package to identify significantly related biological processes, molecular functions, cellular components, and metabolic pathways. The ggplot2 package was then used to visualize the results. Finally, p-values were corrected using the Benjamini and Hochberg method (25). R packages such as “VariantAnnotation”, “MutationalPatterns”, and “maftools” were utilized to analyze the somatic mutation data from TCGA database. This analysis was used to construct mutational landscape maps and to compare genomic variation characteristics between the high- and low-risk groups.

### 2.8 Immune infiltration analysis

The Cell-type Identification By Estimating Relative Subsets Of RNA Transcripts (CIBERSORT) (26) algorithm was implemented to assess the extent of immune cell infiltration, while the LM22 feature set was employed to analyze the ratio of 22 immune cell subpopulations. The single-sample gene set enrichment analysis (ssGSEA) was conducted using the “GSVA” R-package platform (27) to calculate the enrichment scores of immune cell subpopulations, thereby enabling a systematic assessment of the immune infiltration status of the tumor microenvironment (28). The ESTIMATE algorithm (Estimation of Stromal and Immune cells in Malignant Tumor tissues using Expression data) is a systematic approach to evaluating the levels of stromal and immune cell infiltration in tumor tissues. It achieves this by integrating the expression data of specific molecular markers. The algorithm outputs quantitative metrics, including stromal score, immune score, ultimate score, and tumor purity, thereby providing a multidimensional quantitative analysis framework for tumor microenvironment studies (29).

### 2.9 Statistical analysis

The statistical charts of this study were primarily produced by the R program, and the Shapiro–Wilk method was employed to assess the normality of the continuous data. The t-test was employed to calculate the differences between groups for all data sets that met the normal distribution, while the Wilcoxon test was used to calculate the differences between groups for data sets that did not meet the normal distribution. Pearson’s correlation analysis was performed to identify the lncRNAs that exhibited a strong correlation with MPT, and one-way Cox regression analysis was employed to identify the prognosis-related genes. Subsequently, LASSO-Cox regression analysis was employed to construct a risk prognostic model. The diagnostic predictive efficacy of the screened genes in hepatocellular carcinoma was evaluated using the subject ROC curves calculated using the pROC package. The Kaplan–Meier survival analysis was applied to evaluate the prognostic predictive value of MPT-driven necrosis-associated lncRNAs. To address this challenge, the false discovery rate (FDR) was controlled through the implementation of the Benjamini-Hochberg (BH) test correction, which was used to adjust the p-values of GO/KEGG and differential expression analysis. The degree of association between gene sets and enriched functions or processes was examined by hypergeometric distribution in GO and KEGG analyses. The prognostic predictive value of the MPT-driven necrosis-associated lncRNA prognostic risk score column line plot was evaluated through the application of a calibration curve and decision curve analysis. The ESTIMATE score, CIBERSORT immune infiltration analysis, ssGSEA, and drug susceptibility analysis did not conform to the normal distribution using the Wilcoxon rank-sum test. A p-value of less than 0.05 was considered to be a statistically significant difference.

## 3 Results

### 3.1 Identification and screening of MPT-driven necrosis-associated lncRNAs

**Figure 1:**
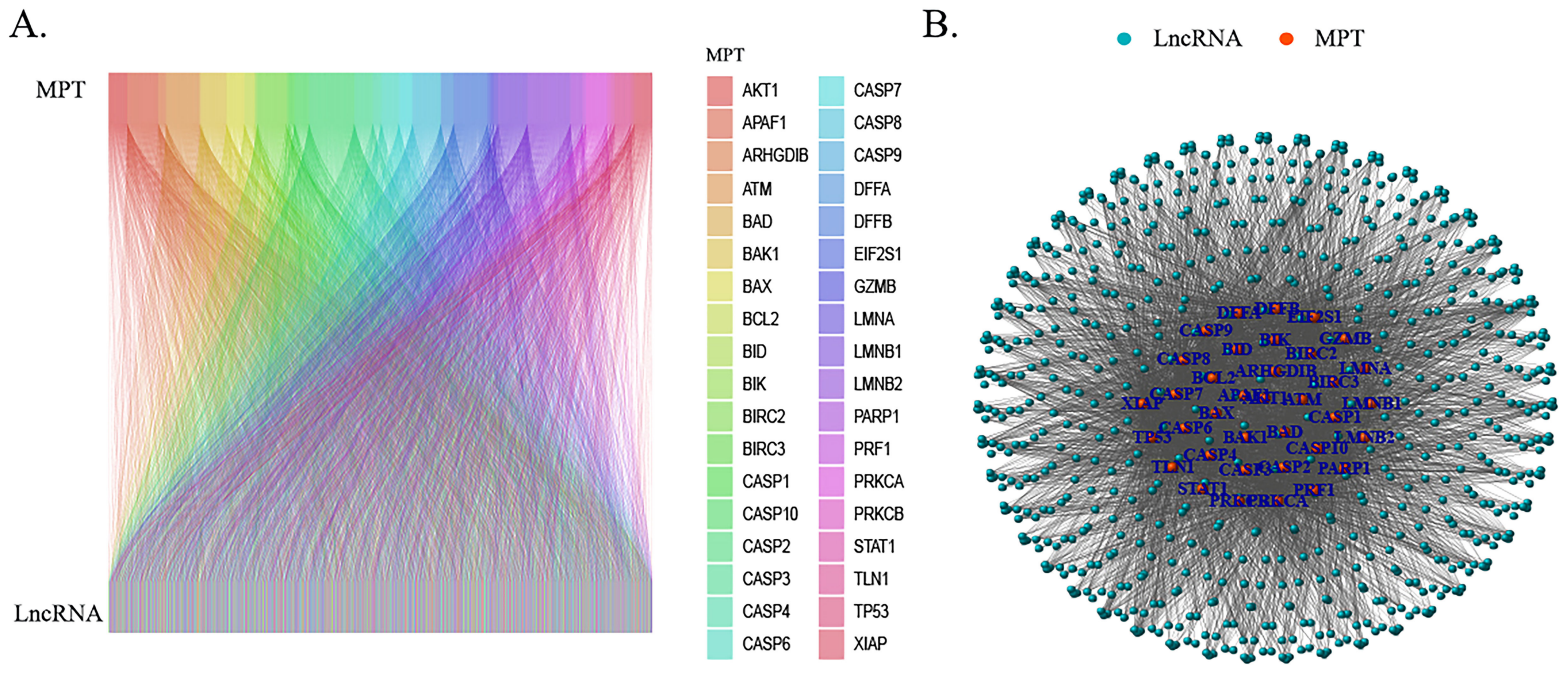
Screening of MPTDNRlncRNAs via Pearson’s correlation analysis. **(A)** Sankey diagram illustrating relationships between MPTDNRGs and MPTDNRlncRNAs. **(B)** Co-expression network mapping interactions between MPTDNRGs and MPTDNRlncRNAs. MPT, mitochondrial permeability transition; MPTDNRGs, mitochondrial permeability transition-driven necroptosis-related genes; MPTDNRlncRNAs, mitochondrial permeability transition-driven necroptosis-related long non-coding RNAs.

The integration of clinical and transcriptomic data from TCGA database, complemented by the exclusion of six patients with incomplete survival records, yielded a comprehensive data set encompassing the clinical and transcriptomic information of 363 patients. A Pearson’s correlation analysis was performed on the obtained lncRNAs and 39 MPT-driven necrosis-associated genes (MPTDNRGs). The results are presented in **Table 2** as MPT-driven necrosis-associated lncRNAs (MPTDNRlncRNAs). The total number of lncRNAs obtained was 1,132. Subsequently, the co-expression network and Sankey diagram of lncRNAs and MPT-driven necrosis-related genes were established, thereby illustrating the relationship between MPTDNRGs and microRNA-targeted long non-coding RNAs (MPTDNRlncRNAs) (**Figures 1A, B**).

**Table 2:** Examples of correlations between MPT-driven necrosis-related genes and lncRNAs.

MPT	LncRNA	Cor	p	Regulation
CASP2	SNHG16	0.448519411803331	1.15713774479855e−19	Positive
BIRC2	SNHG16	0.515526865397815	1.92435128346985e−26	Positive
APAF1	SNHG16	0.409580396439736	2.32972789440126e−16	Positive
EIF2S1	SNHG16	0.470912521593616	9.15826407428389e−22	Positive
PARP1	SNHG16	0.408739580159943	2.7166234869794e−16	Positive
ATM	SNHG16	0.458213697358944	1.48781390276256e−20	Positive
PRKCA	SNHG16	0.464377956488576	3.90069531504992e−21	Positive
LMNB2	SNHG16	0.452033733754437	5.54273211502215e−20	Positive
BAX	SNHG29	0.474464310927791	4.11187787901899e−22	Positive
TP53	SNHG29	0.51290435643141	3.79015742841727e−26	Positive
PARP1	SNHG29	0.43717888932712	1.17491701598278e−18	Positive
LMNB2	SNHG29	0.489176394475642	1.34747039749348e−23	Positive
BAX	TYMSOS	0.44243746555677	4.05435475521464e−19	Positive
PARP1	AL162595.1	0.539417411761677	3.02964274454516e−29	Positive

MPT, mitochondrial permeability transition; lncRNAs, long non-coding RNAs.

### 3.2 Modeling of MPT-driven necrosis-associated lncRNA scoring

**Figure 2:**
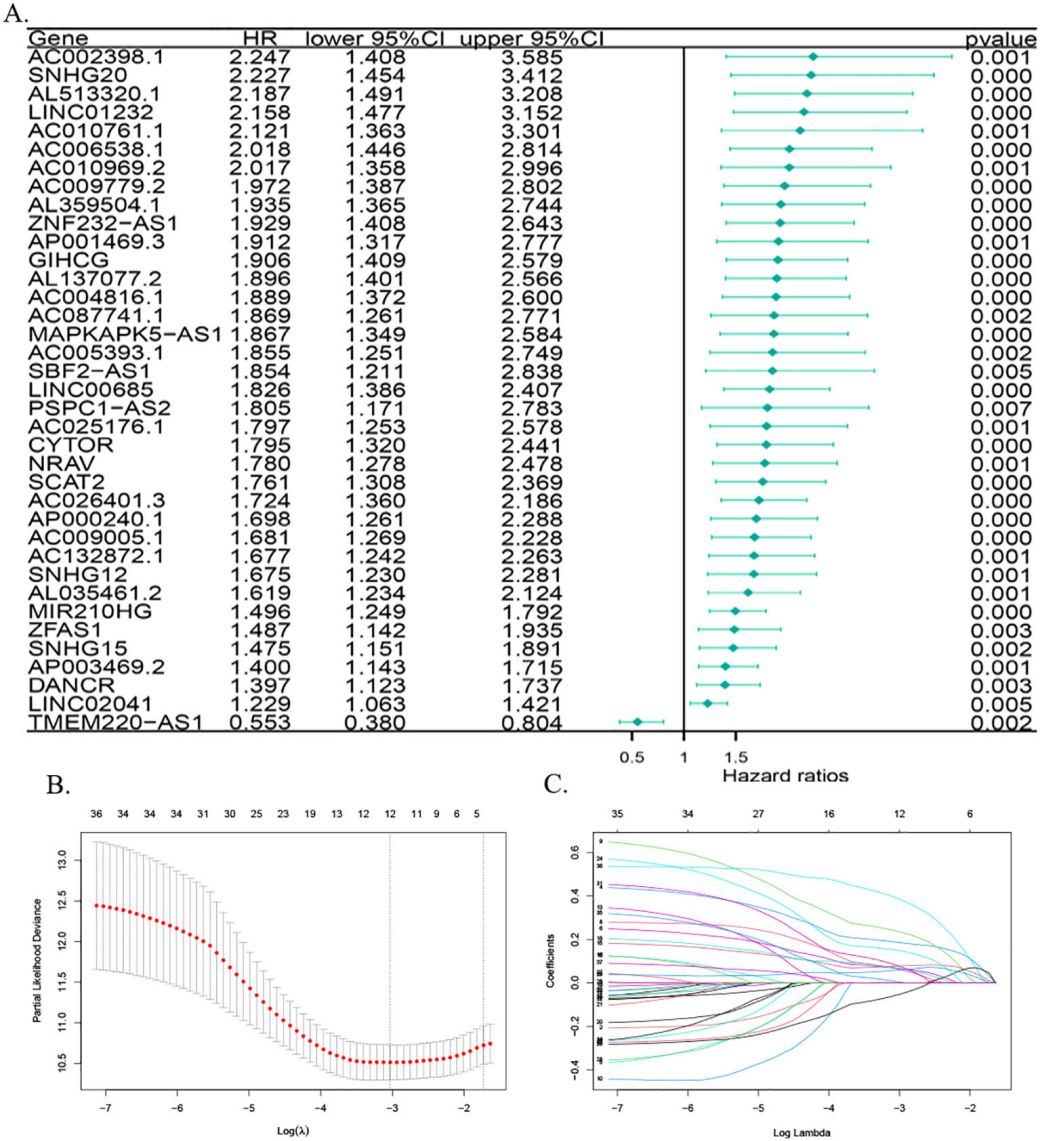
Screening and construction of the MPT-driven necroptosis-related lncRNA prognostic model in HCC. **(A)** Forest plot of lncRNAs associated with HCC prognosis. **(B)** Coefficient path plot: the lower X-axis displays log(λ) values (regularization parameter), while the upper X-axis indicates the number of non-zero coefficient variables at each λ. The Y-axis represents cross-validated deviance, with red dots denoting mean deviance values and vertical bars showing standard error ranges. The left dashed line marks the optimal λ (lambda.min), and the right dashed line corresponds to the model within one standard error of lambda.min (lambda.1se). **(C)** Variable trajectory plot: upper values indicate variable counts at different λ levels, lower values represent log(λ), and left-axis values depict coefficient magnitudes. MPT, mitochondrial permeability transition; HCC, hepatocellular carcinoma; lncRNA, long non-coding RNA.

To further construct the prognostic risk score, patients with HCC were divided into two groups—the training group (n = 182) and the test group (n = 181)—via randomization. Utilizing one-way Cox regression analysis, 37 MPTDNRlncRNAs were identified as being associated with overall survival (OS), as illustrated in the forest plots (**Figure 2A**). Among the 37 lncRNAs, 36 lncRNAs were considered to be strongly associated with poor prognosis of HCC patients (HR > 1), and one lncRNA had a hazard ratio (HR) of less than 1, suggesting that there was a negative correlation between this lncRNA and the prognosis of hepatocellular carcinoma patients. Subsequently, LASSO-Cox regression analysis was employed to identify prognostically relevant MPTDNRlncRNAs, and three MPTDNRlncRNAs (LINC00685, MIR210HG, and GIHCG) were selected to construct a risk score model. **Figures 2B, C** illustrate the outcomes of the two-dimensional visualization and analysis of the regularization process, respectively. The coefficient path diagram (**Figure 2B**) focuses on presenting the progressive contraction trajectory of each variable coefficient with increasing λ values, revealing the dynamic process of feature selection. The variable trajectory diagram (**Figure 2C**), in contrast, visualizes the cluster change patterns and convergence paths of the variable coefficients with different values of λ through parameter space mapping. The risk score of HCC patients was calculated using the following formula: risk score = LINC00685 * 0.0312677536891744 + MIR210HG * 0.00937471222523869 + GIHCG * 0.045196328584586.

### 3.3 Evaluation of predictive efficacy of MPT-driven necroptosis-related lncRNAs in HCC

**Figure 3:**
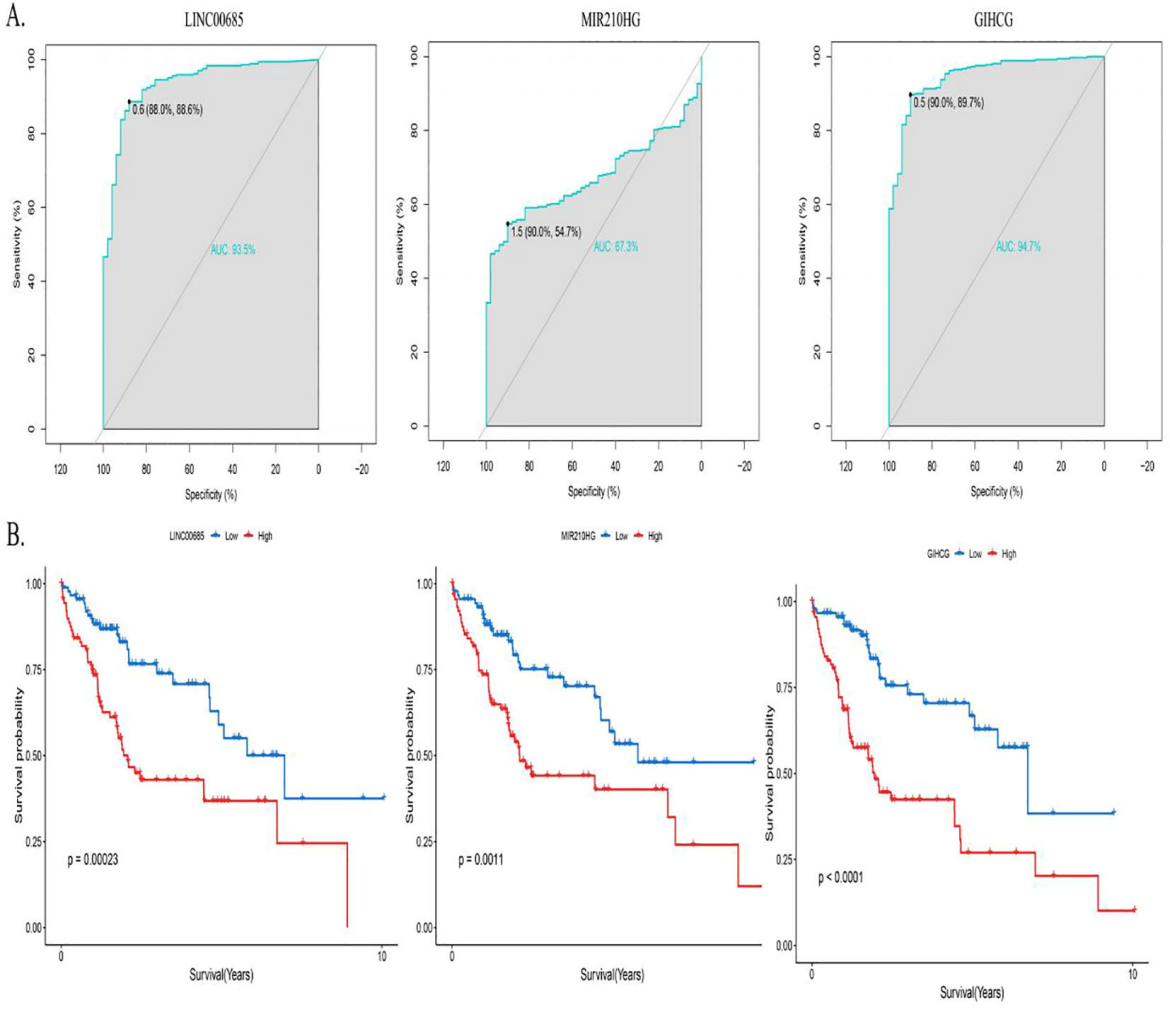
Predictive performance of prognostic genes. **(A)** ROC curves assessing diagnostic accuracy of prognostic lncRNAs in HCC. **(B)** Kaplan–Meier survival curves demonstrating OS differences between high- and low-expression groups. ROC, receiver operating characteristic; lncRNA, long non-coding RNA; AUC, area under the curve; OS, overall survival.

ROC curves were plotted for the diagnosis of hepatocellular carcinoma patients using MPT-driven necrosis-associated lncRNAs. The AUC was calculated for LINC00685, MIR210HG, and GIHCG to be 0.935, 0.673, and 0.947, respectively. This indicates that LINC00685 and GIHCG have high diagnostic values for hepatocellular carcinoma patients (**Figure 3A**). The Kaplan–Meier survival curves were used to perform survival analysis of patients, and it was found that the OS of patients with high expression of LINC00685, MIR210HG, and GIHCG was significantly lower than that of patients with low expression, and this difference was statistically significant (all p < 0.05) (**Figure 3B**).

### 3.4 MPT-driven necroptosis-related lncRNA expression and validation

**Figure 4:**
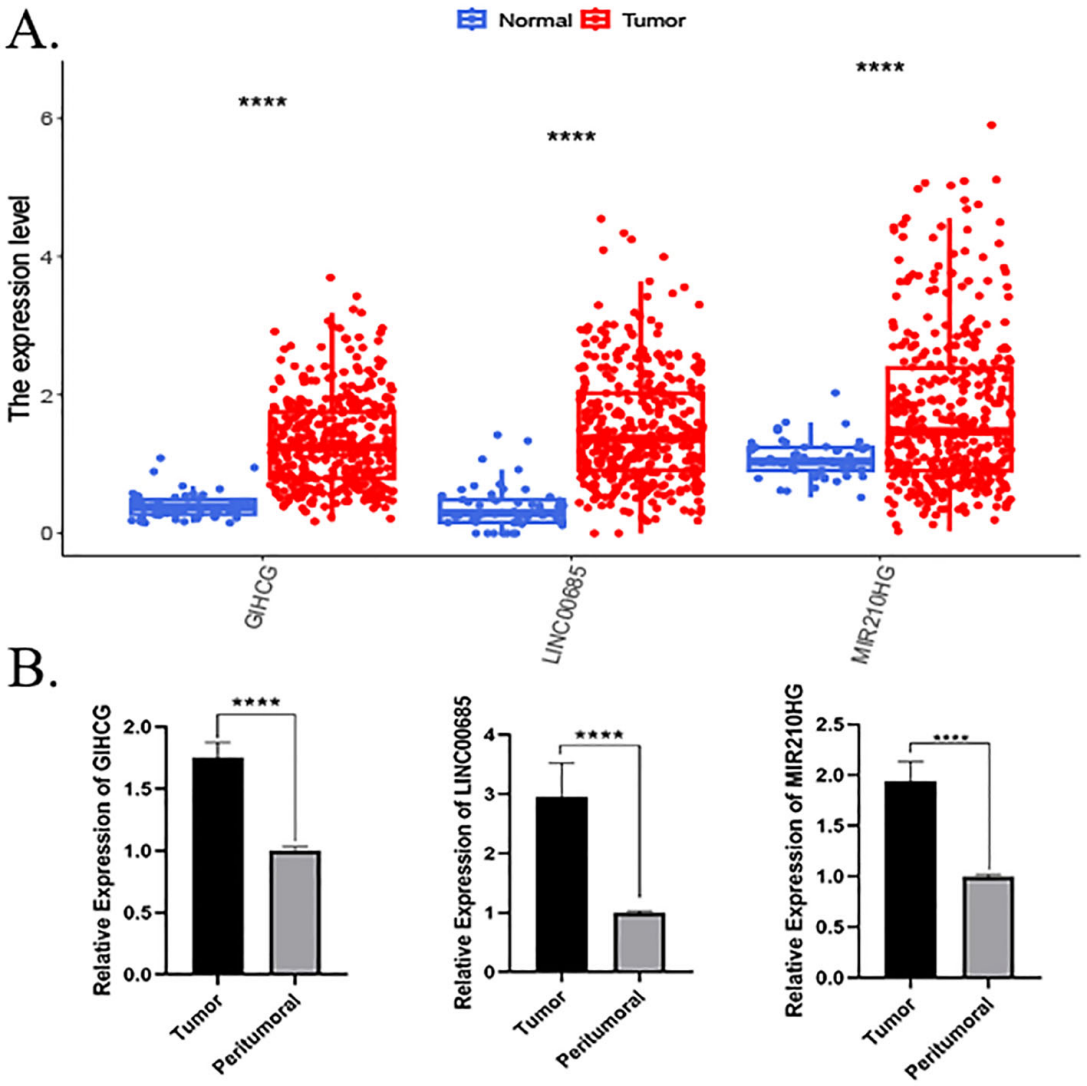
Expression validation of prognostic genes. **(A)** Differential expression of prognostic lncRNAs in HCC versus normal tissues (TCGA database). **(B)** RT-qPCR validation of LINC00685, GIHCG, and MIR210HG expression in clinical HCC samples. p < 0.05, *p < 0.01, **p < 0.001, and ***p < 0.0001; ns, not significant. lncRNA, long non-coding RNA; HCC, hepatocellular carcinoma; TCGA, The Cancer Genome Atlas; RT-qPCR, real-time quantitative polymerase chain reaction.

A comparative analysis of the lncRNA expression levels in HCC and normal patients, as recorded in TCGA database, revealed that the expression levels of LINC00685, GIHCG, and MIR210HG were significantly elevated in HCC tissues compared to normal liver tissues (**Figure 4A**). Concurrently, we obtained cancerous and paraneoplastic tissues from HCC patients who underwent hepatectomy at our hospital. We then examined the expression levels of three MPTDNRlncRNAs. The results demonstrated that the expression levels of LINC00685, GIHCG, and MIR210HG were significantly elevated in HCC tissues (**Figure 4B**).

### 3.5 Prognostic value of the MPT-driven necroptosis-related lncRNA risk score

**Figure 5:**
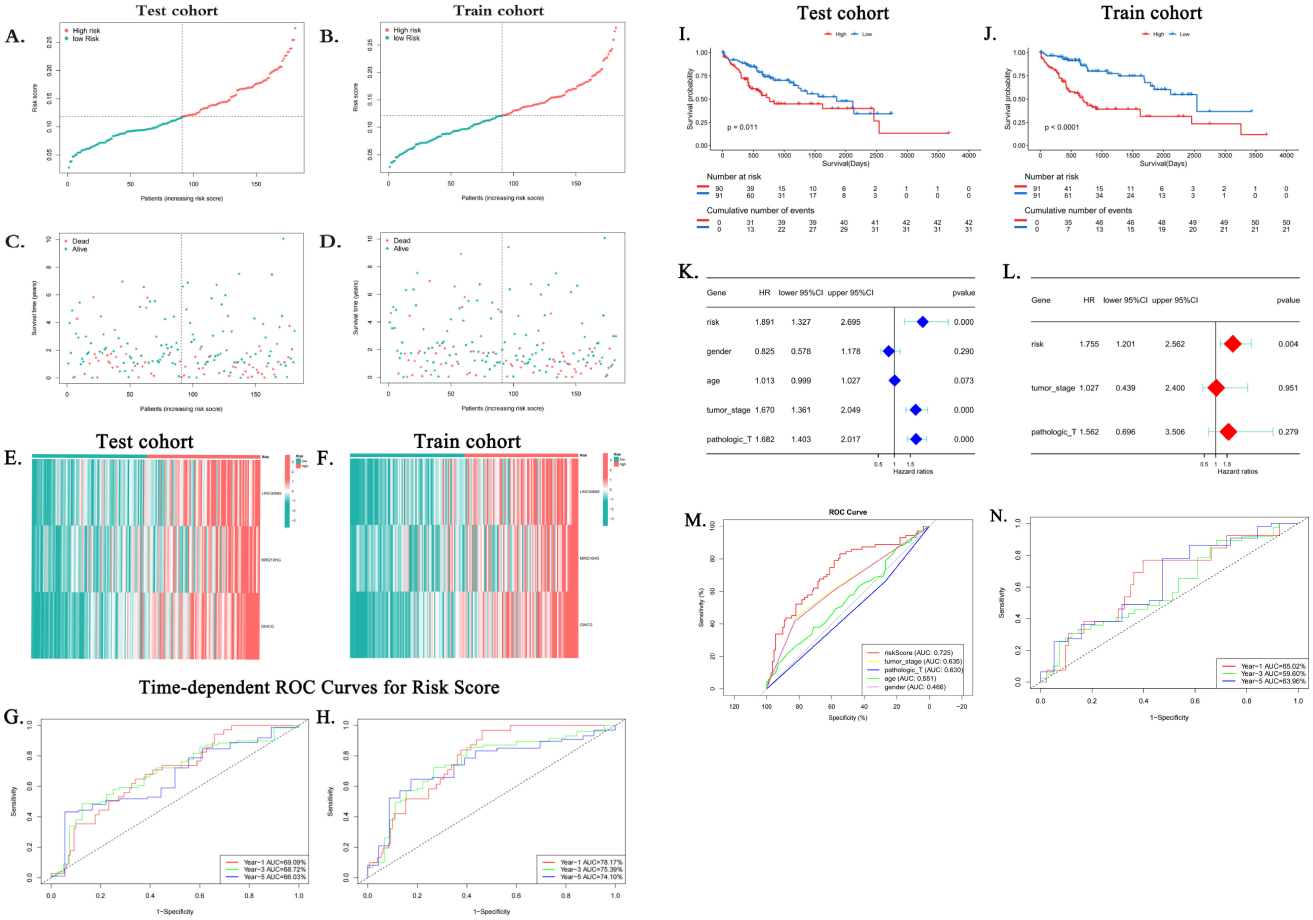
Clinical utility of the risk score. **(A–D)** Distribution of risk scores and survival status in training/test cohorts. **(E, F)** Risk heatmaps: as risk scores increase, the expression level of lncRNAs increases. **(G, H)** Time-adjusted ROC curves for the prediction of 1-, 3-, and 5-year survival of training and validation groups. **(I, J)** Kaplan–Meier survival curves show a decrease in survival rates over time for high-risk subgroups (p < 0.05). **(K, L)** Forest plots from the clinicopathologic variables and the risk score in both the univariate and multivariate Cox regression analyses. **(M)** ROC curves comparing risk score versus clinical variables (age, sex, grade, and stage). AUC, area under the curve; HCC, hepatocellular carcinoma; MPTDNRlncRNAs, MPT-driven necroptosis-related long non-coding RNAs; lncRNA, long non-coding RNA; ROC, receiver operating characteristic. **(N)** ROC curves of the risk model in the ICGC validation cohort (n = 98). The AUC values were 65.02% (1-year survival), 59.60% (3-year survival), and 63.96% (5-year survival).

In accordance with the risk scoring method delineated in the preceding section, risk scores were computed for all samples. The median of the resulting risk score was then employed as a threshold value to further categorize the patients in the training and test groups into the high-risk and low-risk groups. Scatter plots of the relationship between risk score calculated from the risk score and prognostic survival status demonstrated that the number of patients who died correspondingly increased as the risk score increased in both the training group and the test group. This result was consistent with the model’s expectations (**Figures 5A–D**). In the risk heatmap of expression profiles in individual samples, the expression of all three lncRNAs utilized to construct the model was elevated in the high-risk group (**Figures 5E, F**).

Subsequently, time-dependent ROC curves were plotted, and the AUC values of the patients’ 1-, 3-, and 5-year risk scores were calculated. The results demonstrated that in the training group, the AUC values for the 1-, 3-, and 5-year risk scores of HCC patients were 0.7817, 0.7539, and 0.741, respectively (**Figure 5G**). In contrast, in the test group, the AUC values for the 1-, 3-, and 5-year risk scores of HCC patients were 0.6909, 0.6872, and 0.6603, respectively (**Figure 5H**). Subsequently, survival analyses were conducted for patients with high- and low-risk profiles in the training and test groups, respectively. The findings indicated that the overall survival rate of the hepatocellular carcinoma patients in the high-risk category was notably lower than that in the low-risk category within both the training group and the test group. The observed discrepancy was found to be statistically significant (p < 0.05) (**Figures 5I, J**), thereby suggesting that patients with high-risk scores exhibited a more unfavorable prognosis. Univariate and multifactorial Cox regression analyses yielded results suggesting that risk stratification based on risk score is an independent risk factor for hepatocellular carcinoma patients (**Figures 5K, L**). Furthermore, ROC curves were plotted based on clinicopathologic characteristics, age, gender, and risk score. The results demonstrated that the AUC of the risk score (0.725) was superior to each clinical characteristic, including age (0.466), gender (0.551), grading (0.63), and staging (0.635) (**Figure 5M**). This finding indicates that the predictive capability of the risk score surpasses that of the clinical characteristics employed to evaluate patient risk.

#### 3.5.1 External validation in ICGC cohort​

In order to assess the generalizability of the model, its validation was performed in an independent cohort from the International Cancer Genome Consortium (ICGC) database (ICGC-LIRI-fr, n = 98). The application of the risk formula and median cutoffs resulted in the categorization of patients into the high- and low-risk groups. The results of the ROC analysis indicated the high predictive accuracy of the method for 1-year survival (AUC = 0.650), 3-year survival (AUC = 0.596), and 5-year survival (AUC = 0.640) (**Figure 5N**).

### 3.6 Principal component analysis of risk stratification

**Figure 6:**
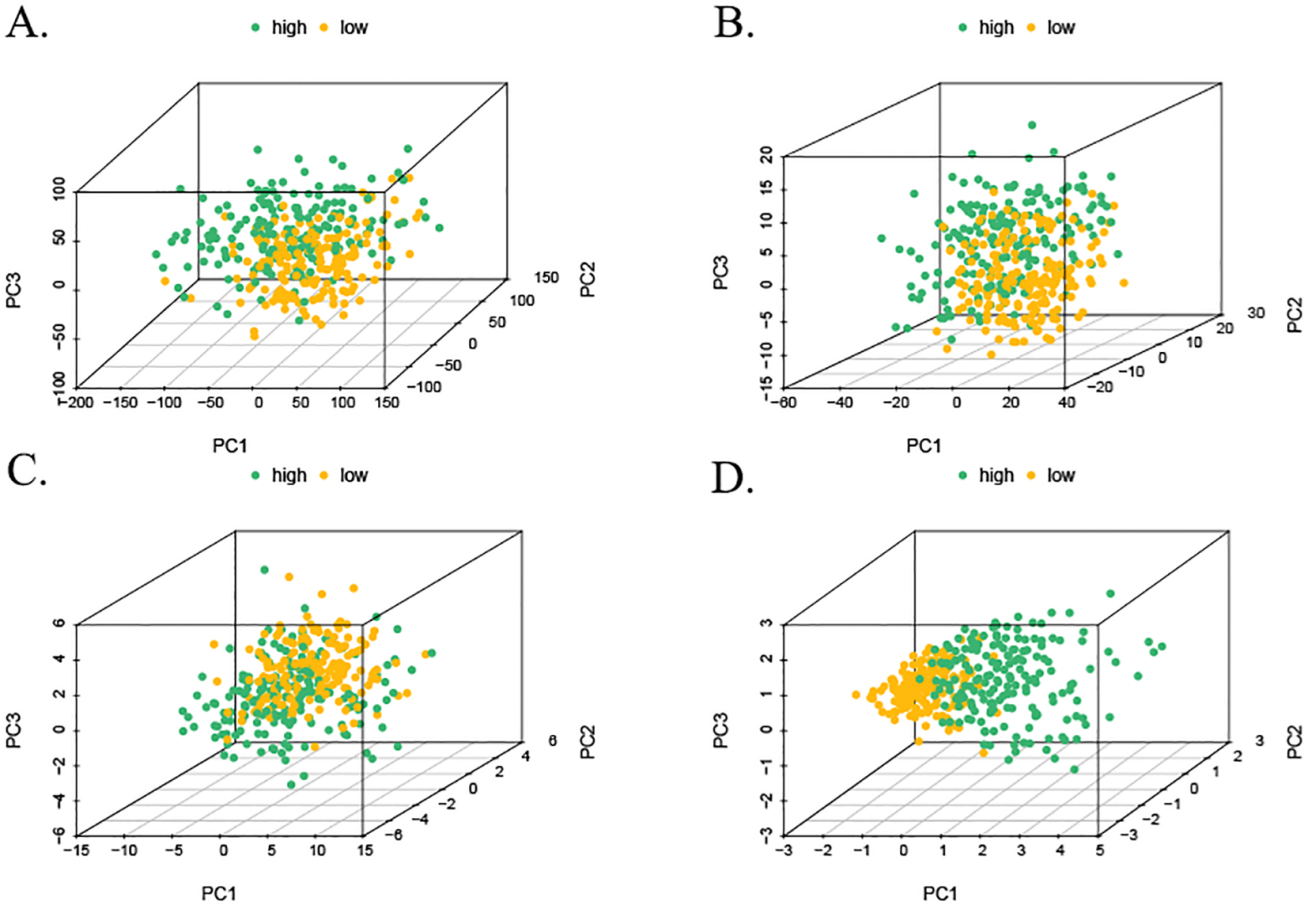
Three-dimensional visualization of PCA. **(A)** PCA of all detected gene expressions. **(B)** PCA of MPTDNRGs. **(C)** PCA of MPTDNRlncRNAs. **(D)** PCA of risk score stratification. PCA, principal component analysis; MPTDNRGs, mitochondrial permeability transition-driven necroptosis-related genes; MPTDNRlncRNAs, mitochondrial permeability transition-driven necroptosis-related long non-coding RNAs.

The samples from the high- and low-risk groups were subjected to PCA downscaling analysis based on the risk scores constructed from the three MPTDNRlncRNAs. The expression of MPT-associated mRNAs, MPTDNRlncRNAs, and all the detected genes was considered. The 3D scatter plots of PCA showed all the gene expression (**Figure 6A**). The expression levels of MPTDNRGs, MPTDNRlncRNAs, and risk score (**Figures 6B–D**) were all found to be significantly correlated with the observed clustering patterns. The risk score group exhibited a particularly pronounced clustering tendency, as evidenced by the statistical significance of the observed differences. The implementation of a more nuanced patient categorization system is imperative.

### 3.7 Prognostic nomogram construction and validation in HCC

**Figure 7:**
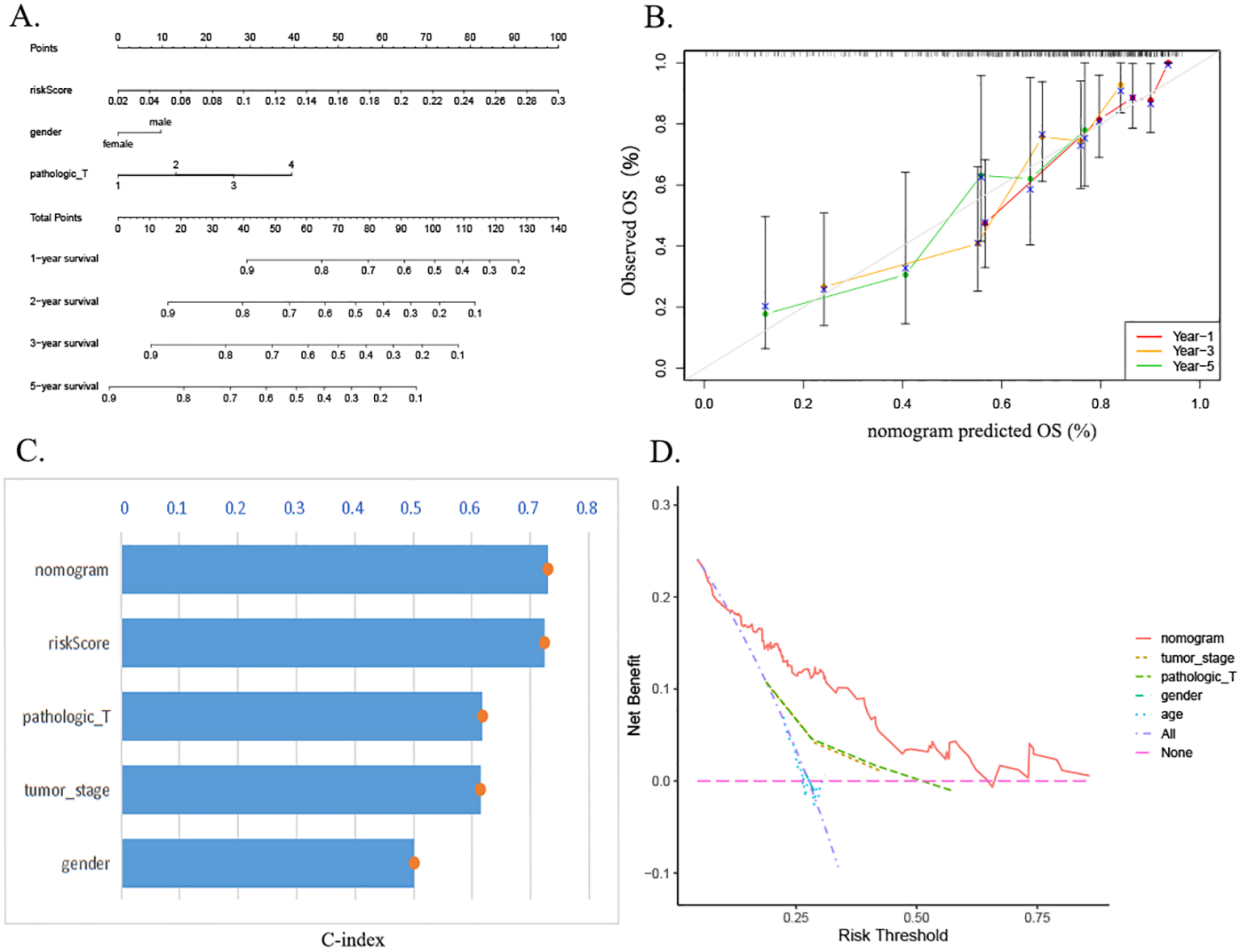
Construction and evaluation of the nomogram. **(A)** Nomogram of the risk prognostic model. **(B)** Calibration curve assessing agreement between predicted and observed outcomes at 1-, 3-, and 5-year intervals. **(C)** The C-index demonstrates superior predictive accuracy of the nomogram compared to other clinical factors. **(D)** DCA evaluating the clinical utility of the nomogram. C-index, concordance index; DCA, decision curve analysis.

The prognostic column-line plots incorporated risk scores, age, and staging (**Figure 7A**). Subsequent calibration curve analysis of the column charts was then conducted to assess the prognostic outcomes of HCC patients at 1, 3, and 5 years after diagnosis. The graph calibration curve results demonstrated that the column-line graphs exhibited substantial agreement in predicting prognosis (**Figure 7B**). Furthermore, the C-index analysis demonstrated that the column-line diagram exhibited superior prognostic accuracy in predicting clinical outcomes in comparison to risk scores, staging, gender, and grading of HCC patients (**Figure 7C**). Additionally, the clinical decision curves revealed that the column-line diagram was more clinically beneficial in comparison to other predictors (**Figure 7D**).

### 3.8 Differential gene function enrichment analysis in high- and low-risk groups

**Figure 8:**
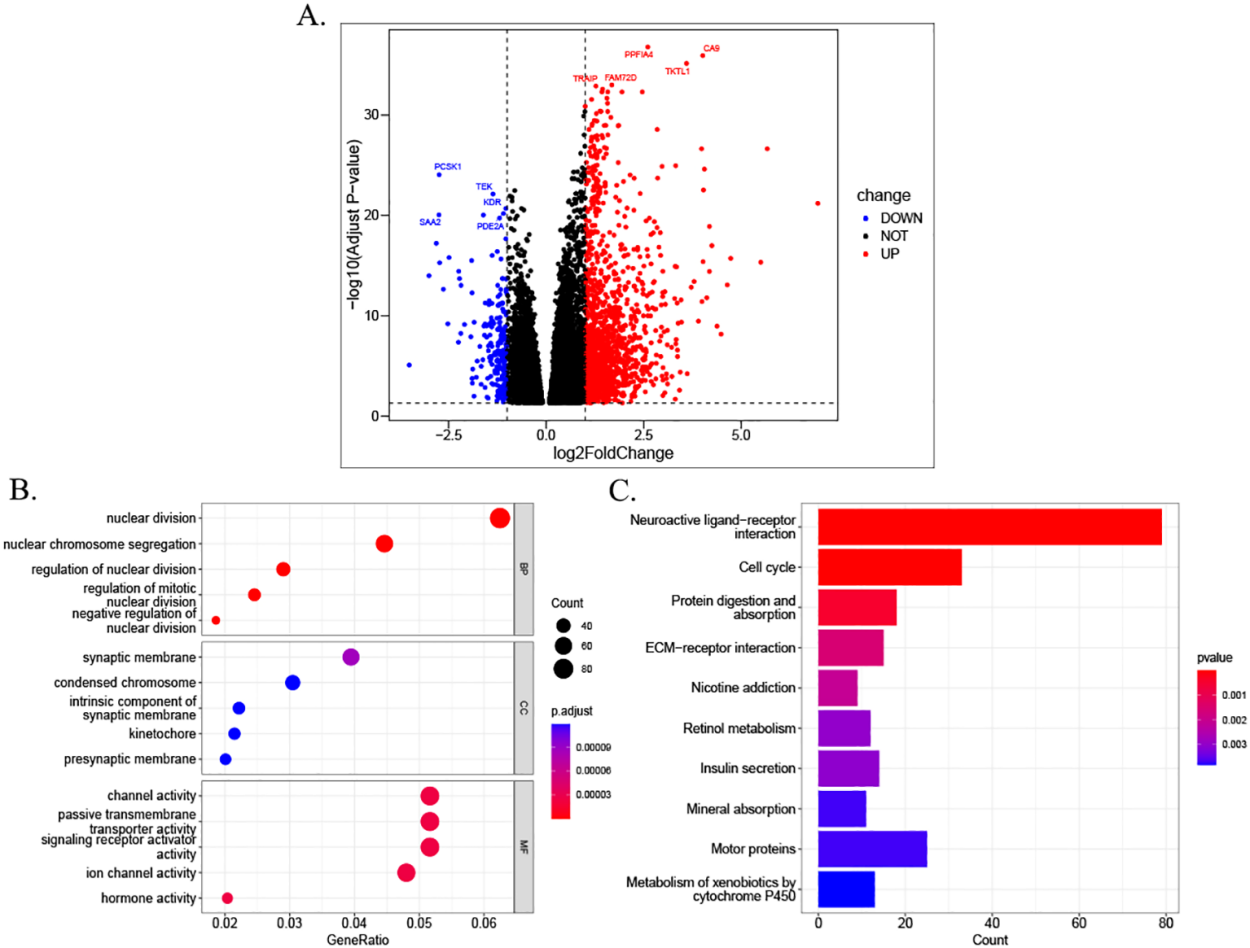
Differential analysis and functional enrichment between risk groups. **(A)** Volcano plot illustrating DEGs between high- and low-risk groups stratified by MPTDNRlncRNA prognostic scores. **(B)** GO enrichment analysis of DEGs in biological processes (BPs), cellular components (CCs), and molecular functions (MFs). Counts indicate the number of enriched genes per GO term; GeneRatio reflects the proportion of enriched genes relative to the total gene set. **(C)** KEGG pathway enrichment results. DEGs, differentially expressed genes; MPTDNRlncRNAs, mitochondrial permeability transition-driven necroptosis-related long non-coding RNAs; GO, Gene Ontology; BP, biological process; CC, cellular component; MF, molecular function; KEGG, Kyoto Encyclopedia of Genes and Genomes.

A total of 1,569 DEGs were identified through a comparative analysis of mRNA expression levels between the high- and low-risk groups of HCC patients in TCGA database. This analysis was conducted based on stringent screening criteria, including a p-value less than 0.05 and a |log2Fold Change| greater than or equal to 1.0 (**Figure 8A**). The study identified 203 downregulated differentially expressed genes and 1,366 upregulated differentially expressed genes. Subsequently, GO enrichment analysis and KEGG enrichment analysis were carried out for these DEGs. The results demonstrated that DEGs in the high- and low-risk groups were significantly enriched in biological process (BP) alterations involving nuclear division, nuclear chromosome segregation, regulation of nuclear division, regulation of nuclear division in mitotic cells, and negative regulation of nuclear division. In molecular function (MF), DEGs were predominantly enriched in channel activity, passive transmembrane transporter activity, signaling receptor activation activity, ion channel activity, and hormone activity. The alterations in cellular components (CCs) manifested predominantly in synaptic membranes, dense chromosomes, intrinsic components of synaptic membranes, mitophagy, and presynaptic membranes (**Figure 8B**). KEGG pathway analysis revealed that DEGs were enriched in neural activity ligand–receptor interactions, cell cycle, protein digestion and absorption, and extracellular matrix (ECM)–receptor interactions (**Figure 8C**).

### 3.9 Analysis of immune cell infiltration in HCC patients

**Figure 9:**
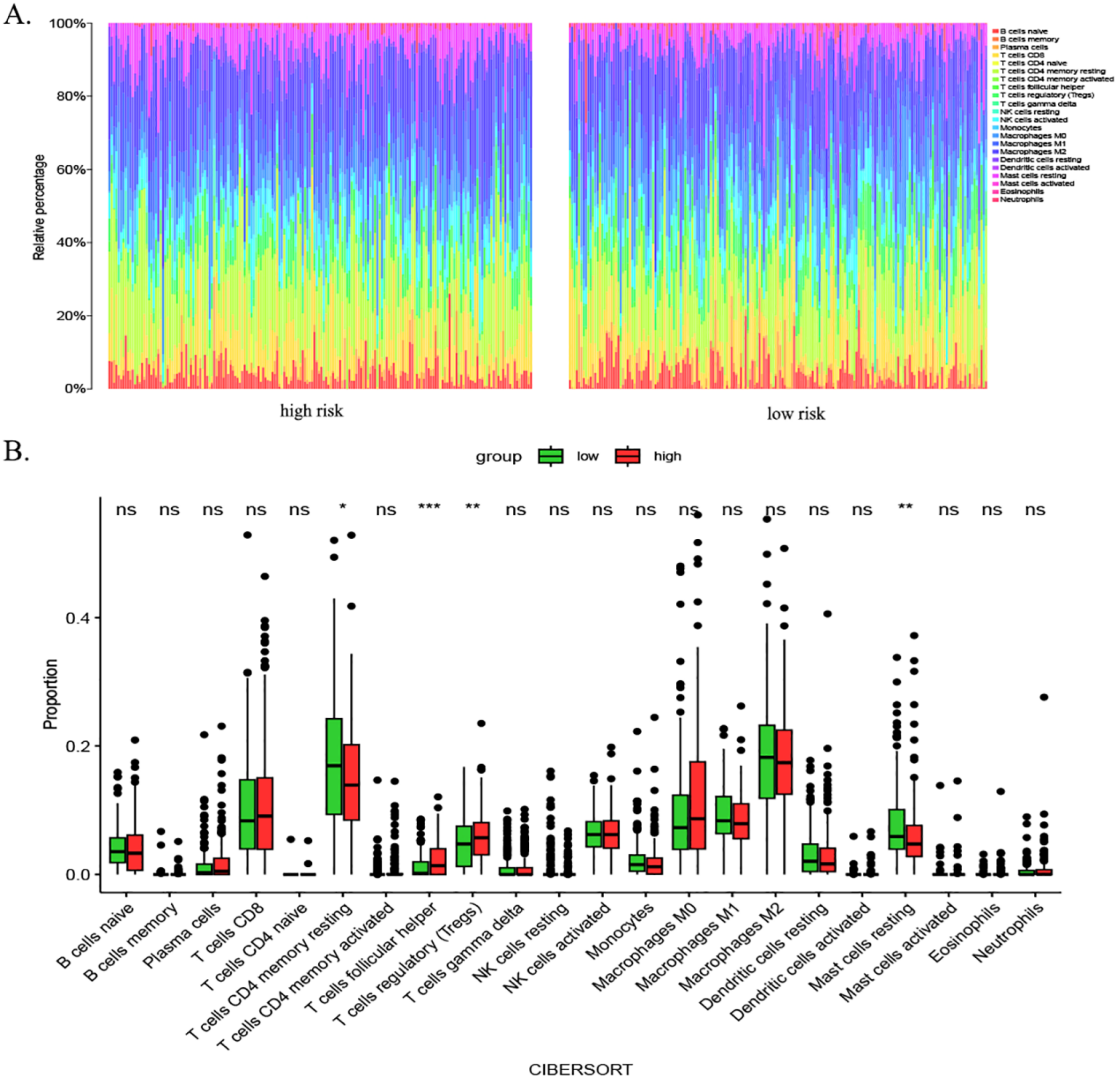
Immune infiltration analysis in HCC patients using CIBERSORT. **(A)** Stacked bar chart illustrating immune cell infiltration profiles in high- and low-risk groups. Distinct colors represent specific immune cell subsets, with bar heights reflecting their relative proportions. This visualization highlights overall similarities in immune infiltration patterns between subgroups. **(B)** Box plots comparing differential infiltration of four immune cell subsets: resting mast cells, Tregs, follicular helper T cells, and resting CD4+ memory T cells (p < 0.05). p-value thresholds: * <0.05, ** <0.01, *** <0.001, and **** <0.0001; ns, not significant. HCC, hepatocellular carcinoma; CIBERSORT, Cell-type Identification By Estimating Relative Subsets Of RNA Transcripts; Tregs, regulatory T cells.

Immune cells play an important role in tumor formation and prognosis. To investigate immune cell infiltration in HCC patients from TCGA cohort, we examined immune cell infiltration in HCC patients from TCGA database. We observed from the immune infiltration stacking histogram that the distribution of infiltrated immune cells was similar between the high- and low-risk subgroups (**Figure 9A**). Analysis based on the CIBERSORT algorithm showed similar distributions of immune cells between the high- and low-risk subgroups in terms of resting mast cells, T regulatory cells, and T follicular helper cells. However, there were differences in the infiltration proportions of four types of immune cells (**Figure 9B**).

### 3.10 Immune infiltration analysis using ssGSEA

**Figure 10:**
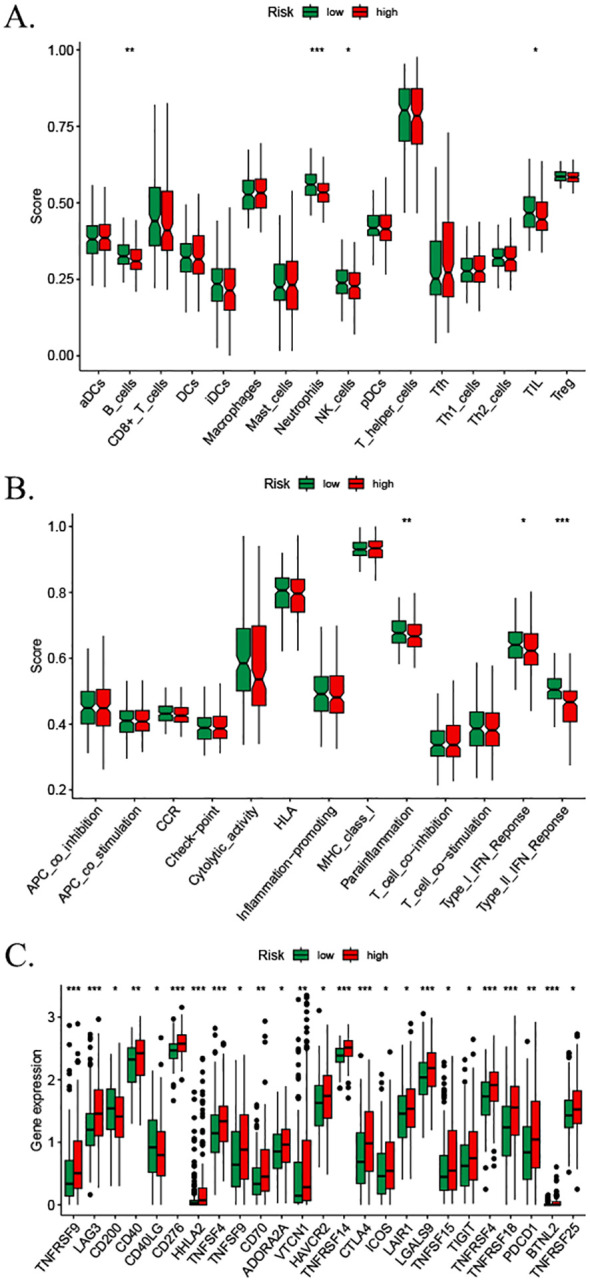
Immune cell infiltration analysis based on ssGSEA scores and differential immune checkpoint expression between risk groups. **(A)** Comparison of immune cell composition between low- and high-risk groups. **(B)** Divergent enrichment of immune function-related pathways in low- versus high-risk subgroups. **(C)** Differential expression of shared immune checkpoint genes between risk groups. p-values: * <0.05, ** <0.01, *** <0.001, and **** <0.0001; ns, not significant. ssGSEA, single-sample gene set enrichment analysis.

We assessed the enrichment level of 16 immune cell associations between the two subgroups using the ssGSEA (30). A subgroup comparison of the proportion of immune infiltration revealed significant differences in B cells, neutrophils, natural killer cells, and immature dendritic cells between the high- and low-risk groups (**Figure 10A**). We then assessed the differences in the enrichment levels of 13 immune function-related pathways between the two groups. The results showed significant differences in parainflammation, type I interferon response, and type II interferon response in the patients in the high- and low-risk groups (**Figure 10B**). Furthermore, we detected significant differences in natural killer cells and immature dendritic cells. We also examined the expression changes of 47 common immune checkpoint genes in the two groups. The results showed the expression levels of 25 immune checkpoints (TNFRSF9, LAG3, CD200, D40, CD40LG, CD276, HHLA2, TNFSF4, TNFSF9, CD70, ADORA2A, VTCN1, HAVCR2, TNFRSF14, CTLA4, ICOS, LAIR1, LGALS9, TNFSF15, TIGIT, TNFRSF4, TNFRSF18, PDCD1, BTNL2, and TNFRSF25) (**Figure 10C**). Discrepancies between the CIBERSORT and ssGSEA stem from fundamental algorithmic differences. Specifically, the CIBERSORT quantifies cell fractions via linear deconvolution, a process that necessitates reference profiles that may lack HCC-specific immune subsets. In contrast, the ssGSEA computes enrichment scores of gene signatures, thereby capturing pathway activity rather than absolute abundance.

### 3.11 ESTIMATE immune infiltration analysis

**Figure 11:**
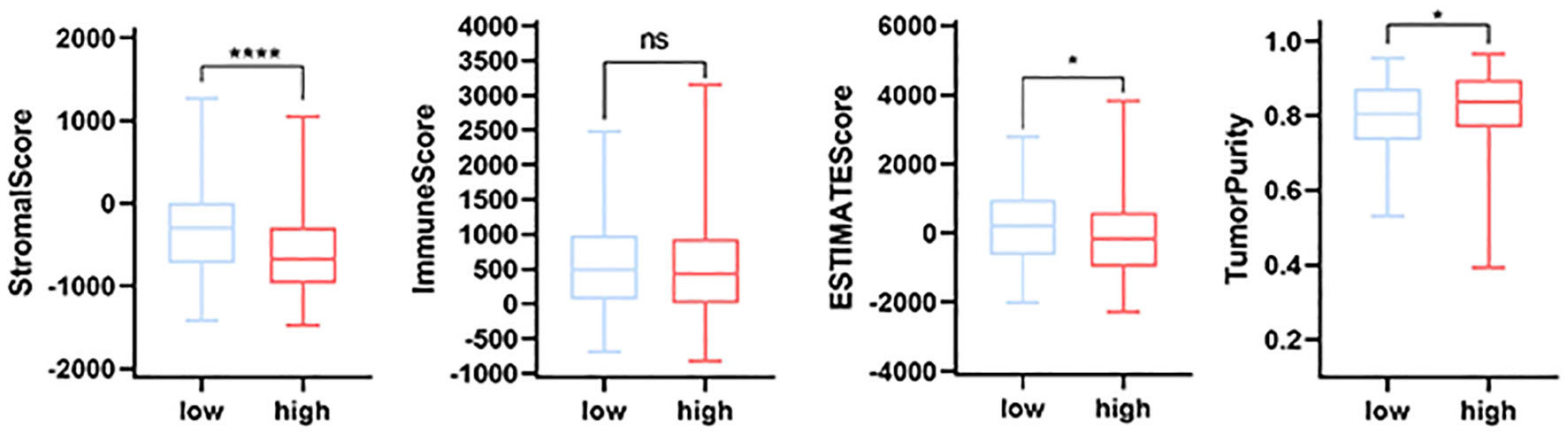
Comparative immune infiltration analysis between high- and low-risk groups using ESTIMATE. p-values: * <0.05, ** <0.01, *** <0.001, and **** <0.0001; ns, not significant. ESTIMATE, Estimation of stromal and immune cells in malignant tumors using expression data.

The estimation of stromal and immune cells in malignant tumor tissues using expression data (ESTIMA) analysis revealed that the patients in the high-risk and low-risk groups of the MPT-driven necrosis-associated lncRNA scoring model exhibited significant disparities in stromal scores, ESTIMA scores, and tumor purity. However, the discrepancy between the two groups was not statistically significant in terms of immune scores (p > 0.05). Specifically, the patients in the high-risk group exhibited lower stromal scores and composite scores, as well as higher tumor purity (**Figure 11**).

### 3.12 Somatic mutation analysis of the MPT-driven necrosis-associated lncRNA risk scoring model

**Figure 12:**
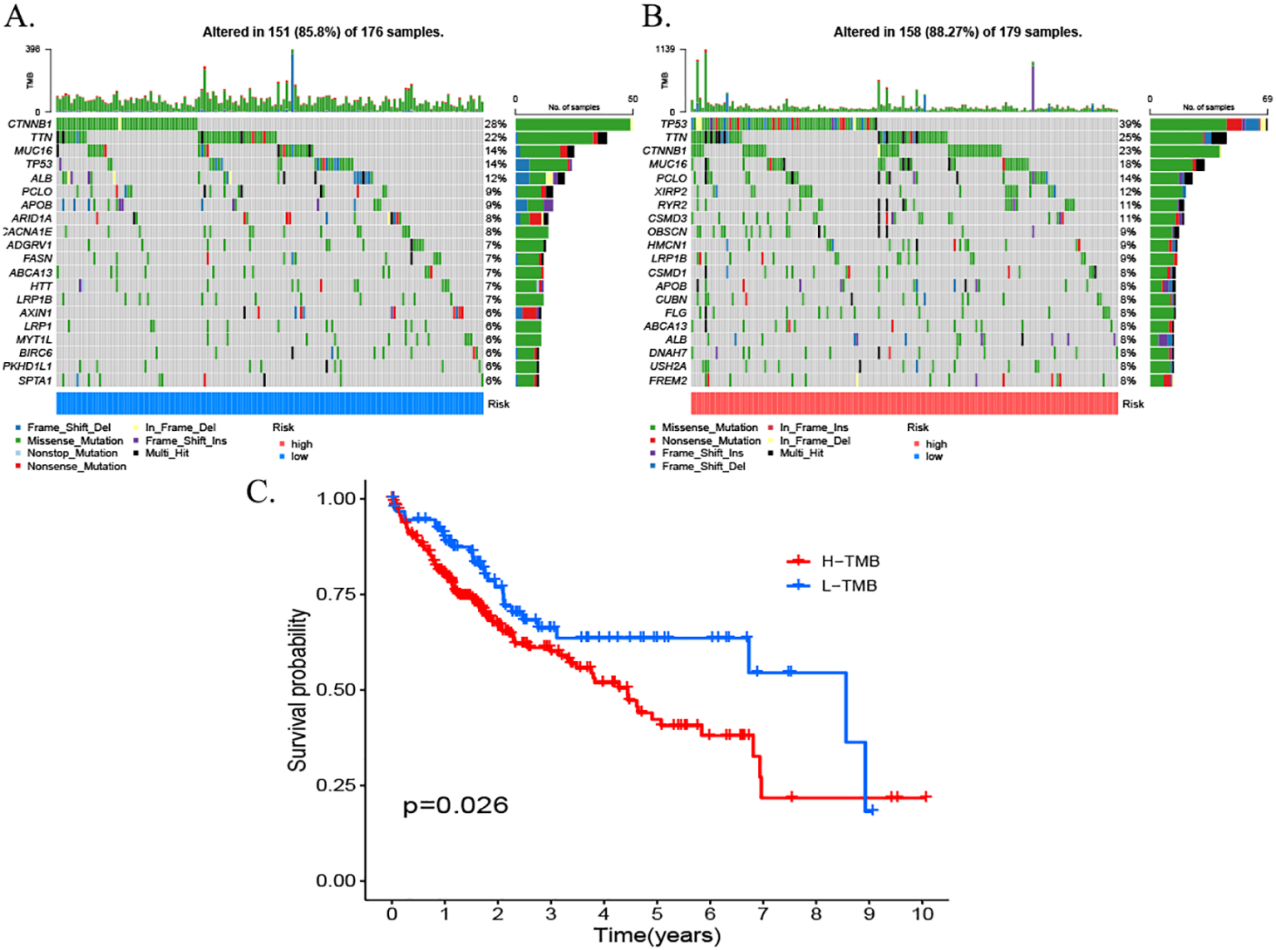
Somatic mutation waterfall plots for the high- and low-risk groups. **(A)** High-risk group. **(B)** Low-risk group. **(C)** Survival rates for patients with high TMB. TMB, tumor mutational burden.

As illustrated by the somatic mutation landscape map, there appears to be a lack of significant discrepancy in the mutation patterns of genes between the high- and low-risk subgroups. The predominant mutation type observed in both groups is missense mutations. The waterfall map further elucidates the 20 genes with the highest mutation frequency in the high- and low-risk groups. The results indicate that TP53 is the gene with the highest mutation frequency in the high-risk scoring group, while CTNNB1 is the gene with the highest mutation frequency in the low-risk scoring group. Furthermore, missense mutations were observed to be the predominant type of mutation in both genes (**Figure 12**).

## 4 Discussion

HCC is a highly aggressive malignancy that affects the gastrointestinal tract, with global incidence and mortality rates that are significant and well-documented, respectively. The therapeutic outcome of this condition is contingent upon the clinical stage at the time of diagnosis. Despite the diversification of liver cancer treatments nowadays, the prognosis of liver cancer remains dismal due to its heterogeneity. Biomarkers play an essential role in the treatment of various tumors, particularly in predicting drug efficacy and monitoring disease progression. In addition to conventional peripheral blood biomarkers, such as AFP, the correlation between PD-L1 expression level, tumor mutational burden (TMB), and other factors, and patient responsiveness to immunotherapy has emerged as a significant research focus. Furthermore, investigations are underway to identify changes in gene expression profiles, circulating tumor DNA, and free DNA. The objective of these investigations is to ascertain biomarkers that can accurately predict patient prognosis (31, 32). Mitochondria, the cell’s primary respiratory factories, have been shown to generate Reactive Oxygen Species (ROS), which have the potential to influence tumorigenesis and progression through various pathways, including DNA damage and lipid peroxidation (33, 34). MPT has been identified as a critical factor in the development of various types of tumors, including HCC and breast cancer. However, there is a paucity of research on the application of MPTDNRlncRNAs in HCC. In this study, LINC00685, GIHCG, and MIR210HG were selected to establish a prognostic risk scoring model for MPTDNRlncRNAs in HCC. This model was developed using various methods, including Pearson’s correlation and LASSO regression analysis. The diagnostic and prognostic predictive abilities of the three genes in question were ascertained through the analysis of survival curves and ROC curves. As demonstrated in prior studies, elevated levels of LINC00685 expression have been observed to be associated with unfavorable prognoses in cases of HCC. In particular, the suppression of LINC00685 expression has been shown to markedly reduce the proliferation, invasion, and migration capabilities of HCC cells (35). GIHCG, a recently identified lncRNA, is located on the human chromosome 12q14. It has been demonstrated that lncRNA GIHCG is expressed abnormally in a wide range of tumors and is associated with prognosis and drug resistance (36). In addition, studies have shown that elevated expression levels of lncRNA GIHCG in HCC tissues may promote HCC cell proliferation and invasive migration with the help of regulatory miR-429 (37). Furthermore, lncRNA GIHCG has been associated with poor prognosis of HCC (38, 39). The occurrence and prognosis of a variety of cancers have been associated with the regulation and expression of MIR210HG (40–44). High MIR210HG expression has been found to correlate with advanced HCC clinical stage, large tumor size, microvascular infiltration, and unfavorable histological differentiation. Moreover, high MIR210HG expression has been identified as an independent adverse prognostic factor affecting overall survival. *In vitro* investigations further demonstrated that the silencing of MIR210HG impeded the proliferation, migration, and invasion of HCC cells, thereby functioning as an oncogenic lncRNA (45). These findings indicate that the prognostic genes identified in this study can effectively predict the clinical outcomes of HCC patients. Moreover, *in vitro* validation experiments revealed that these three genes exhibited elevated expression levels in HCC. Therefore, the aforementioned results validate the reliability of the model and indicate that these three MPTDNRlncRNAs may play an important role in HCC. Consequently, these three MPTDNRlncRNAs can provide reliable biomarkers for the diagnosis and treatment of HCC.

The study associated a high-risk score with poorer HCC prognosis, as confirmed by clinical correlation analysis. To validate the 3-MPTDNRlncRNA risk score, patients were stratified into the low- and high-risk groups using the median score. Time-dependent ROC curves demonstrated significant associations between the risk score, clinical characteristics, and prognosis. The risk score showed robust predictive accuracy, with training cohort AUCs of 0.782 (1-year survival), 0.754 (3-year survival), and 0.741 (5-year survival) and test cohort AUCs of 0.691 (1-year survival), 0.687 (3-year survival), and 0.660 (5-year survival). ROC analyses further revealed superior predictive capability versus clinicopathologic features. Critically, univariate and multivariate analyses established risk stratification as an independent prognostic factor for HCC. These results confirm the clinical utility of this MPT-associated lncRNA risk model for guiding personalized HCC management (46).

In the context of ESTIMA analysis, the risk score demonstrated a negative correlation with stromal score and composite score and a positive correlation with tumor purity. In consideration of these findings, a hypothesis can be proposed that posits immune infiltration as a potential significant factor in the prognosis of HCC, influenced by risk modeling. Notably, our somatic mutation analysis (**Figure 12**) yielded complementary genomic insights into the biological heterogeneity captured by the risk score model. The waterfall plots disclosed distinct mutational profiles between the high- and low-risk groups. A salient finding was the predominance of TP53 missense mutations within the high-risk cohort. This observation is consistent with the well-established function of TP53 dysfunction in instigating genomic instability, aggressive tumor behavior, and poor prognosis in HCC. In contrast, CTNNB1 missense mutations were predominantly observed in the low-risk group. CTNNB1 mutations have been shown to induce the constitutive activation of the Wnt/β-catenin signaling pathway, which is associated with a specific subclass of HCC that may exhibit distinct clinical behaviors and potentially a better prognosis compared to TP53-mutant tumors. The differential enrichment of these hallmark driver mutations (TP53 in the high-risk group and CTNNB1 in the low-risk group) strongly supports the biological relevance and stratification capability of the MPTDNRlncRNA-based risk model, as it effectively segregates patients harboring distinct oncogenic drivers linked to known prognostic subgroups. This genomic distinction further underscores the model’s potential utility in identifying patients with differing underlying tumor biology and clinical outcomes. The model demonstrated consistent predictive accuracy in an independent ICGC cohort.

In the field of medicine, nomograms represent a prevalent clinical instrument that facilitates predictions for patients through a combination of intuitive and straightforward presentation. In this study, a column-line diagram containing risk score, age, and T-stage was established by combining clinical information. The good prognostic predictive value of the high-risk score was further verified by calibration curves, decision curve analysis (DCA) plots, and C-index. In accordance with the findings of the GO and KEGG enrichment analyses, the high- and low-risk groups were predominantly enriched in the cell cycle of biological processes. The tumor mutation load waterfall map demonstrated that CTNNB1 missense mutations were predominant in the low-risk group, whereas TP53 missense mutations were predominant in the high-risk group, which is consistent with the results of a previous study (47). The genetic alterations, specifically the mutations, in the genes TP53 and CTNNB1 have been demonstrated to be a causative factor in the maintenance of telomeres, the P53 pathway, and the Wnt/β-catenin signaling pathway. These alterations, in turn, have been shown to induce numerous other abnormal changes, affecting the cell cycle and, consequently, leading to HCC.

The tumor microenvironment (TME) is composed of three major components: the extracellular matrix, stromal cells, and infiltrating immune cells. These components have been linked to tumor proliferation, metastasis, and immunosuppression (48). To further explore the mechanism between this risk scoring model and HCC, immune infiltration analysis was performed in this study. However, the CIBERSORT immune infiltration results demonstrated no statistically significant differences in the distribution of immune cells among the different risk groups. Despite the *in silico* analyses suggesting an association between risk scores and immune dysregulation—for example, the ssGSEA showing impaired IFN response in the high-risk group—the absence of experimental validation precludes causal claims. The discordant results obtained from the CIBERSORT analysis and ssGSEA underscore the limitations of extrapolating bulk RNA-seq data to cellular composition. However, the present study found that the HCC patients in the high-risk group had higher levels of T-cell follicular helper cells and regulatory T cells. As indicated by earlier reports, an increased number and active function of Tregs in the TME are closely related to tumorigenesis, progression, and immune escape. These cells are considered to be an important target for tumor immunotherapy (49). T follicular helper cells may play a protective role in non-lymphoid tumors and correlate with an improved clinical response. Although the activation of T follicular helper (Tfh) cells may represent a novel approach, mounting evidence suggests that the expression of Tfh cell markers is associated with various types of cancers (50). These findings further substantiate the conclusions of the present study. The model demonstrated consistent predictive accuracy in an independent ICGC cohort, as evidenced by an AUC of 0.68 for 1-year survival outcomes. This finding reinforces the clinical relevance of the model. This external validation serves to mitigate concerns regarding overfitting and supports the model’s utility in diverse patient populations. Although the findings have been validated in both TCGA and ICGC cohorts, the necessity of prospective multicenter studies is indicated to assess their real-world performance.

Subsequently, we proceeded to analyze the correlation between additional risk scores and immune cells, immune function, and immune checkpoints. In this study, B cells, neutrophils, natural killer cells, and immature dendritic cells were found to be more enriched in the high-risk group than in the low-risk group. Previous studies have shown that a variety of tumors have high levels of resting natural killer cells and reduced plasma cells and neutrophils (51). These results align with the findings of our study, providing further substantiation for the validity of this risk score. The patients in the low-risk group exhibited enrichment in immune function-related pathways, including parainflammatory response, type I interferon response, and type II interferon response. This phenomenon may suggest a correlation between the favorable prognosis of the low-risk group and the activation of the immune system. In recent years, significant advancements have been made in the field of immunotherapy, particularly in the treatment of HCC. These advancements have led to a notable increase in the survival rate of patients diagnosed with this condition (52–54). Consequently, an analysis was conducted to examine the disparities in the expression of immune checkpoints LAG3, CD200, and CD276, which exhibited elevated expression levels in the high-risk group. These observations suggest that patients within this high-risk category may potentially benefit from therapeutic interventions targeting anti-TNFRSF9, LAG3, CD200, and CD276 antibodies. In the context of ESTIMA analysis (55), the risk score exhibited a negative correlation with stromal score and composite score and a positive correlation with tumor purity. In light of these findings, a hypothesis can be formulated positing that immune infiltration may have a significant impact on the prognosis of HCC, as influenced by risk modeling.

This study has limitations. First, the prognostic models rely solely on public database data and lack prospective clinical validation. Second, while we investigated associations between the TME immune cells and the MPTDNRlncRNA risk signature, the precise underlying mechanisms and specific functional roles require further experimental validation.

## 5 Conclusion

Bioinformatics analysis was employed to construct a model of MPT-driven necrosis-associated lncRNA risk score. In addition, the prognostic genes constituting the MPT-driven necrosis-associated lncRNA risk score were explored and verified by various methods. It has been demonstrated that these possess a degree of clinical utility in predicting the prognosis of HCC. It is anticipated that the three prognostic genes will serve as potential biomarkers in future studies of HCC, thereby providing a framework for guiding the diagnosis and treatment of patients (56).

The majority of contemporary HCC prognostic models is predicated on inflammation-related genes, metabolic reprogramming, or clinical parameters (e.g., TNM staging and Child–Pugh score). This model is pioneering in its inclusion of necrosis-associated lncRNAs driven by MPT as a core marker (57). MPT affects cell death and the immune microenvironment by modulating mitochondrial (58) membrane potential. It is strongly associated with drug resistance and immune escape, especially in HBV-associated HCC. In comparison with single-dimensional lncRNA models (e.g., ceRNA network or epigenetic regulation models), this study constructed a multidimensional prognostic model by integrating TCGA transcriptome data, immune infiltration analysis, and *in vitro* experimental validation. The AUC values of the model reached 0.78 and 0.69 in the training set and test set, respectively, which were superior to those of some previously published lncRNA models (e.g., AUC = 0.68 for the metabolism-associated lncRNA-based model of Zheng et al., 2021). Concurrently, the present model disclosed discrepancies in the Tregs and Tfh, along with elevated expression of immune checkpoint genes (e.g., LAG3 and CD276) in the high-risk group. This feature is consistent with the immunosuppressive microenvironment characteristics identified in recent studies, suggesting that the model can identify subpopulations that may benefit from treatment with immune checkpoint inhibitors. In contrast, the majority of existing models (e.g., those based on AFP or genomic instability markers) lacks the capacity to predict response to immunotherapy.

## Data Availability

The datasets presented in this study can be found in online repositories. The names of the repository/repositories and accession number(s) can be found in the article/supplementary material.
